# Assessing harbour porpoise carcasses potentially subjected to grey seal predation

**DOI:** 10.1038/s41598-020-73258-y

**Published:** 2020-10-01

**Authors:** Abbo van Neer, Stephanie Gross, Tina Kesselring, Miguel L. Grilo, Eva Ludes-Wehrmeister, Giulia Roncon, Ursula Siebert

**Affiliations:** 1 Institute for Terrestrial and Aquatic Wildlife Research (ITAW), University of Veterinary Medicine Hannover, Foundation, Werftstraße 6, 25761 Büsum, Germany; 2 Present Address: CIISA - Centre for Interdisciplinary Research in Animal Health, University of Lisbon, Lisbon, Portugal; 3 Present Address: Institute for Marine and Antarctic Studies, University of Tasmania, Hobart, Australia

**Keywords:** Behavioural ecology, Animal behaviour

## Abstract

As a follow-up on the data presented for seals, we herein report and discuss outcomes resulting from a retrospective evaluation of harbour porpoise stranding and necropsy data from Schleswig–Holstein, Germany (n = 4463) to enable an objective evaluation of potential ecological effects of grey seal predation on porpoises. Results are compared to a recent case of definite grey seal predation as well as to reports from other countries. Porpoise carcasses potentially subject to grey seal predation show severe lacerations, with large parts of skin and underlying tissue being detached from the body. Loss of blubber tissue is common. Based on the occurrence frequencies of encountered lesions, a list of parameters as well as a complementary decision tree are suggested to be used for future assessments. The results shown add to an increasingly standardised assessment protocol of suspected grey seal predation cases making respective results comparable between different areas and countries. The usage of a standardised protocol may increase the awareness of grey seal predation and the reporting of such cases. By this, differences in the predation and feeding patterns as well as the potential ecological relevance of this behaviour may be elucidated.

## Introduction

Since the first scientific publication introducing the hypothesis that grey seals (*Halichoerus grypus*) utilise harbour porpoises (*Phocoena phocoena*) as a prey resource^1^, several publications have proven the hypothesis to be true^2–8^. Furthermore, it was shown that this behaviour is not restricted to specific regions but potentially occurs throughout the North Sea and beyond^9^, with not only porpoises but also harbour and grey seals being utilised as prey^10–12^.

Published descriptions of gross pathological examinations of porpoise carcasses include trauma-induced lesions with large areas of detached skin and blubber, canine puncture wounds throughout the lesions and parallel bite and scratch marks in the skin^1,2,6,13^. Based on these observations, a retrospective analysis of the Dutch stranding database indicated that at least 17% of stranded porpoises were likely to have died as a result of grey seal predation, making it one of the most frequent causes of death^13^. In other areas of the world, predation rates of porpoises are largely unknown and therefore the ecological significance of this behaviour is still not entirely clear.

An objective differentiation between lesions induced by grey seals in comparison to other sources of trauma is often difficult. Thus, the situation in porpoises is comparable to the one in seals^14^.

To allow for a comparison of predation rates in different areas, standardised assessment criteria should be used. Therefore, the aim of the present study is to summarise the grey seal predation-related findings collected during necropsies of harbour porpoises found dead between 1990 and 2018 on the coasts of Schleswig–Holstein, Germany. Results are discussed in comparison to a definite case of predation as well as to cases reported in the literature and other origins of trauma such as scavenging or predation by foxes (*Vulpes vulpes*).

## Results

Between 1990 and the end of 2018, data on 4463 harbour porpoise carcasses were recorded in the necropsy and stranding database of Schleswig–Holstein, at the Institute for Terrestrial and Aquatic Wildlife Research (ITAW), University of Veterinary Medicine Hannover, Germany and are available for a retrospective evaluation. Of these 4463 cases, 1183 carcasses showed lesions consistent with trauma and were assessed accordingly. Of the 1183 cases, 933 were categorised as “unknown” and excluded from further consideration due to a lack of sufficiently detailed information or due to advanced decomposition. Of the remaining 250 cases, four were categorised as “observation only” and excluded from any pathologically based assessment, leaving 246 cases including the one definite case from 2017.

### Gross pathological examination

**Figure 1:**
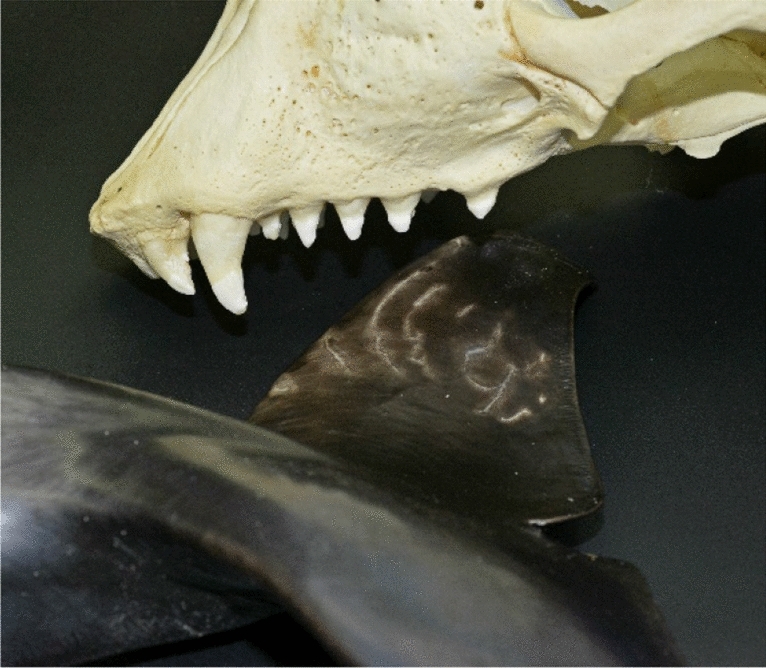
Healed lesions on a porpoise fluke potentially induced by grey seal teeth.

During necropsies of suspected cases, several wound patterns were recorded. The most characteristic wounds in harbour porpoises induced by grey seals are multiple large lacerations with a smooth, linear and cut-like wound margin. Large parts of the skin and blubber are usually detached from the underlying tissue and either still partly attached as skin flaps or fully removed. Manipulation of the skin and blubber tissue by teeth and claws is evident and often repetitive; parallel situated puncture and scratch induced lesions can be found. Besides cases for which grey seal predation was regarded as the most likely direct cause of death, nine cases were identified which had died from other causes but showed different amounts of healed lesions evident as scars. Detected scar patterns resembled for example lesions induced by teeth or scratching with the claws (e.g. Fig. 1).

Using these and further findings, a catalogue of common parameters to be used during macroscopic assessment of porpoise carcasses has been assembled and was updated based on any new findings and experience gathered (see Table S1 in the supplementary material for a detailed description of all parameters).

**Figure 2:**
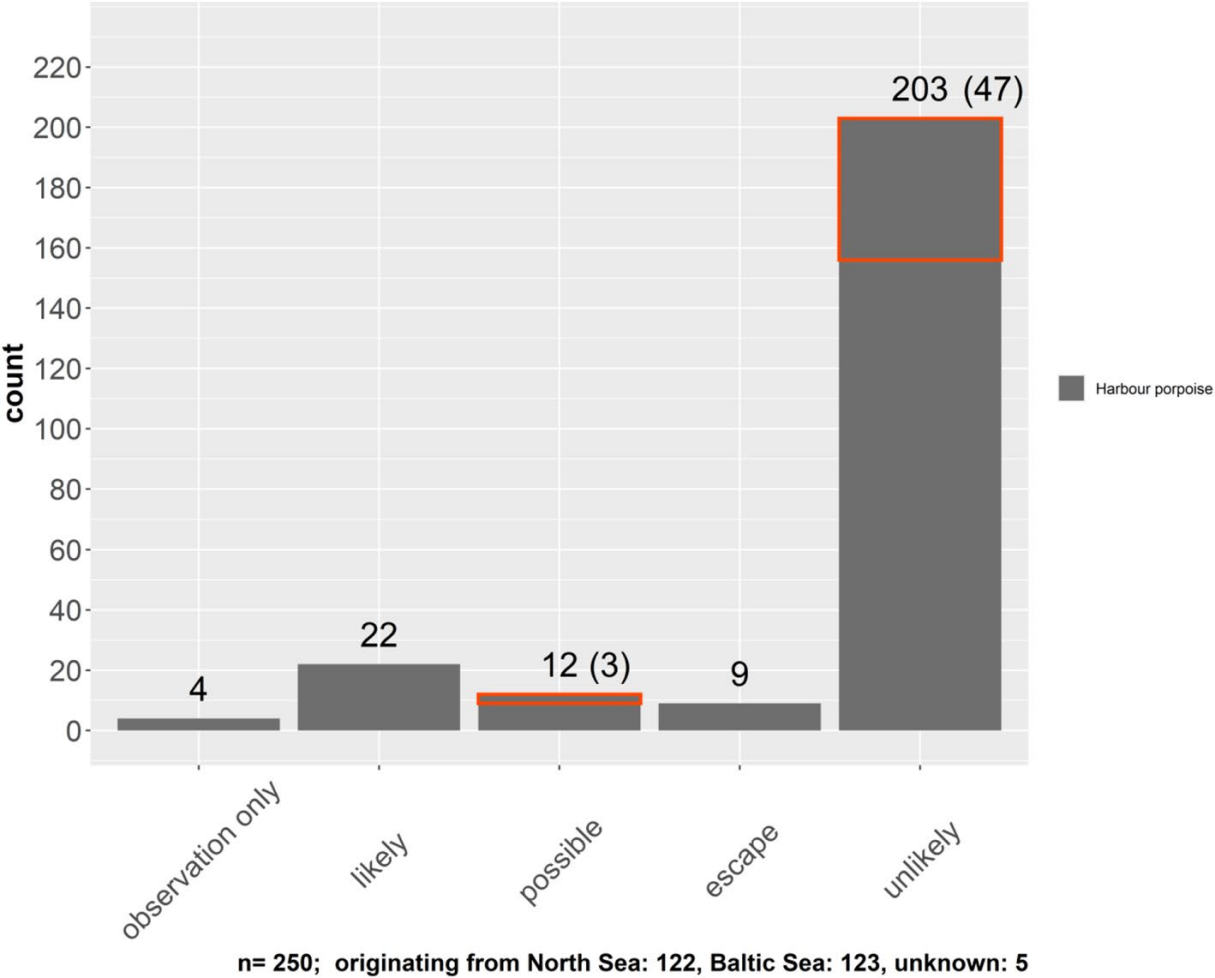
Number of suspected grey seal predation and foxrelated cases of porpoises for the years 1990–2018; categorised by likelihood of grey seal predation (“likely”, “possible”, “unlikely”) and cases that were only observed but without recovery of the carcass (“observation only”).Cases related to fox interaction were included in the category “unlikely” and “possible” in terms of grey seal predation and are highlighted by a red square. The number is shown in brackets. The definite case is included as “likely” in this figure.

**Figure 3:**
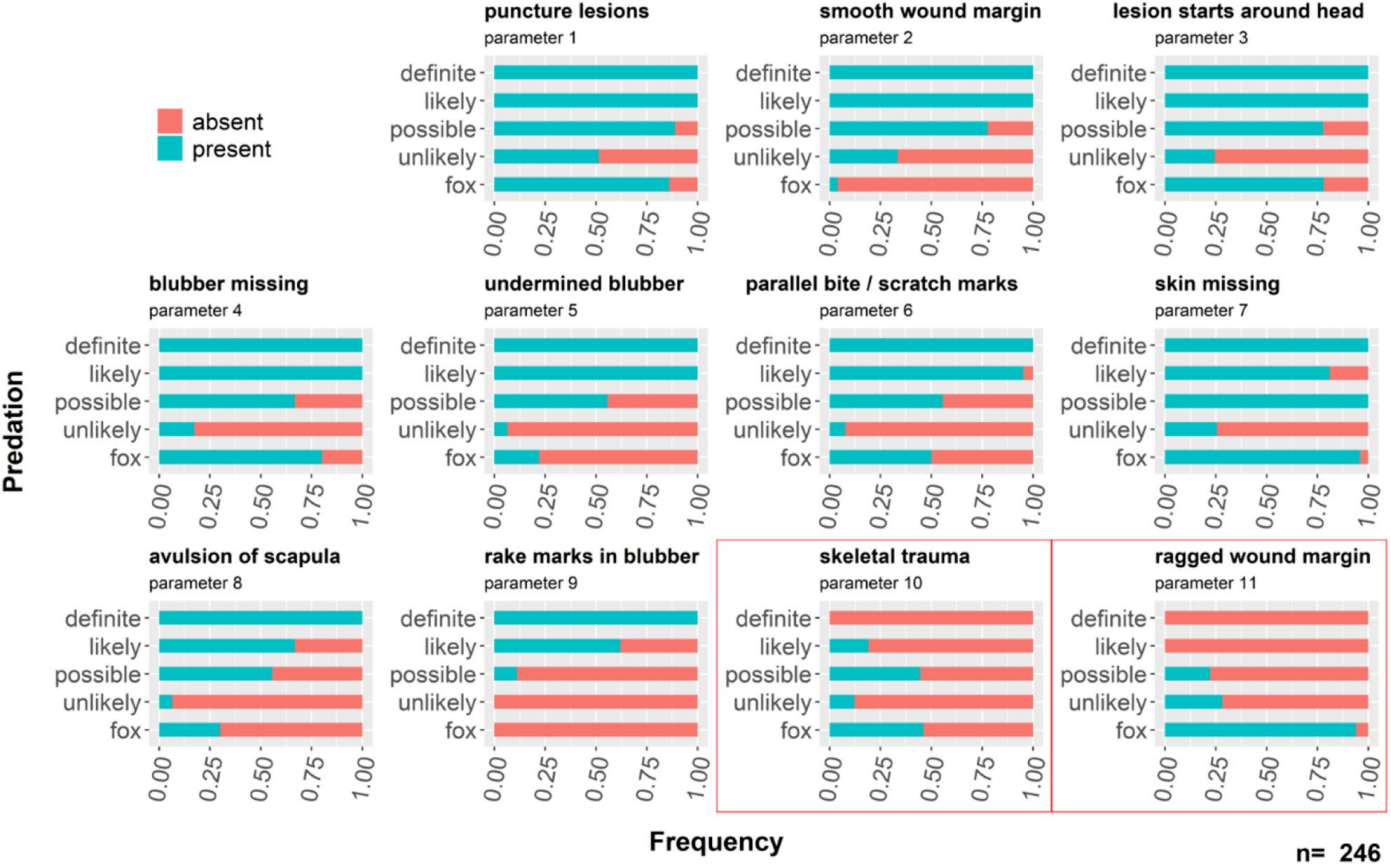
Percentage of occurrence of the 11 parameters in the different categories for suspected grey seal predation cases (“definite”, “likely”, “possible”, “unlikely”) and for suspected fox interactions (“fox”). Cases related to fox interaction are only shown in the category “fox”, despite also being “unlikely” with regards to grey seal predation. Parameters framed with a red rectangle are indicative of an interaction with a fox.

Using the absence and presence of the described parameters as well as the observational reports, 250 cases of suspected grey seal predation were categorised (for the definition of categories, refer to Table 1) (Fig. 2). Categorising, solely based on pathological assessment, was conducted for 246 cases (Fig. 3). All recorded cases of likely grey seal predation originate from the North Sea. To date, no case has been found from the Baltic Sea.

**Table 1:** Categories and their respective description used for rating the likelihood of grey seal predation and fox interaction.

Category	Definition
Definite	The attack was observed and the carcass was retrieved straight after or the origin of trauma could be verified using genetic methods
Likely	It is highly likely that the documented trauma is the result of grey seal predation; the majority of parameters have been found
Possible	It is possible that the documented trauma is the result of grey seal predation; some of the parameters have been found; potentially some indication of a different origin of trauma
Escape	Scar tissue is present, indicating previous interactions with a grey seal, but the cause of death is not directly related
Unlikely	It is unlikely that the documented trauma is the result of grey seal predation; the majority of parameters have not been found; clear indication of a different origin of trauma
Fox	It is unlikely that the documented trauma is the result of grey seal predation, but indicators of an interaction with a red fox have been found

### Decision tree

**Figure 4:**
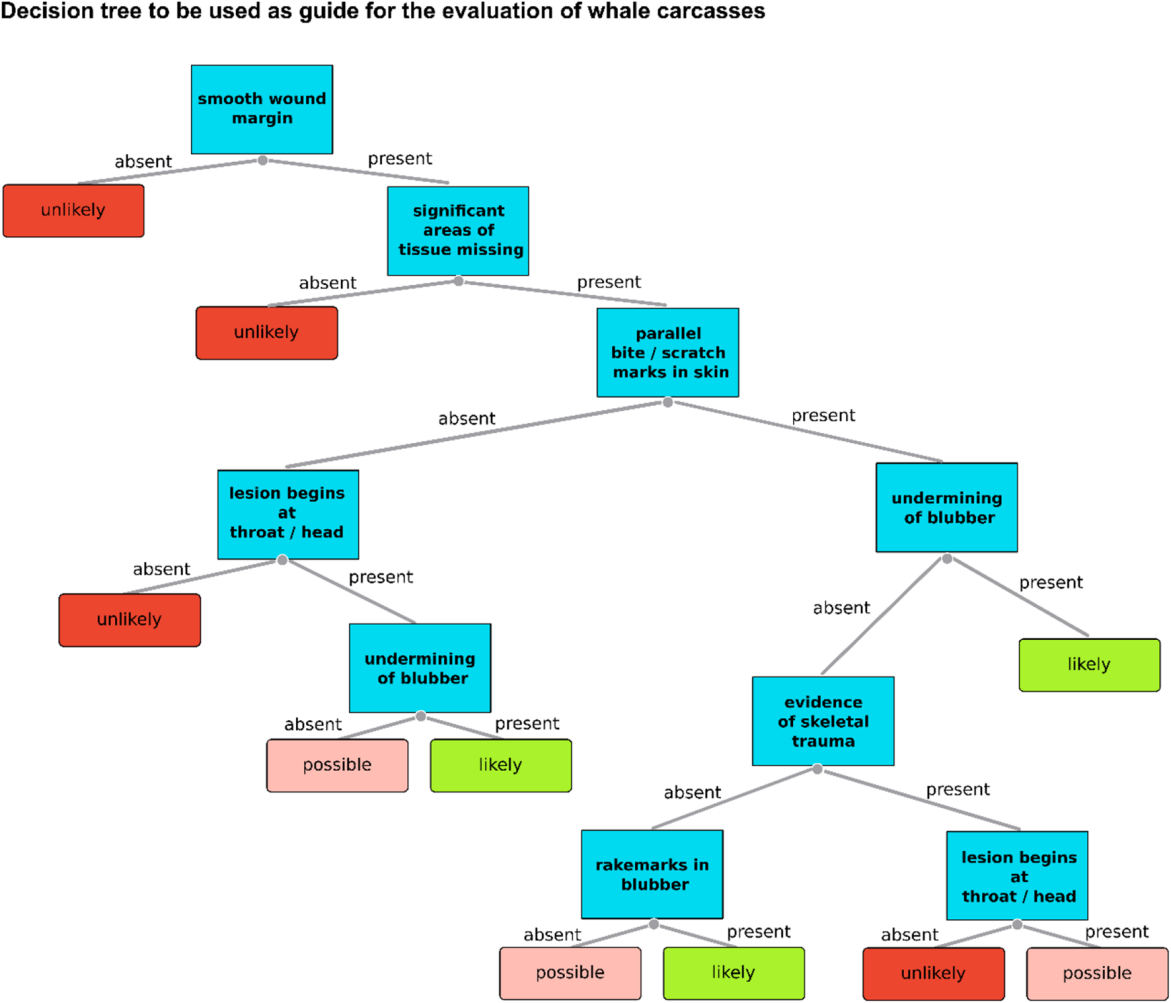
Decision tree developed using the occurrence and combination of detected lesions in porpoise carcasses. Resulting categories indicate the likelihood that the detected lesions are the result of grey seal predation.

As described for seals by van Neer et al.^14^, we developed and verified a decision tree to aid the assessment of harbour porpoise carcasses, using the occurrence as well as the combination of parameters suggested above (Fig. 4).

**Figure 5:**
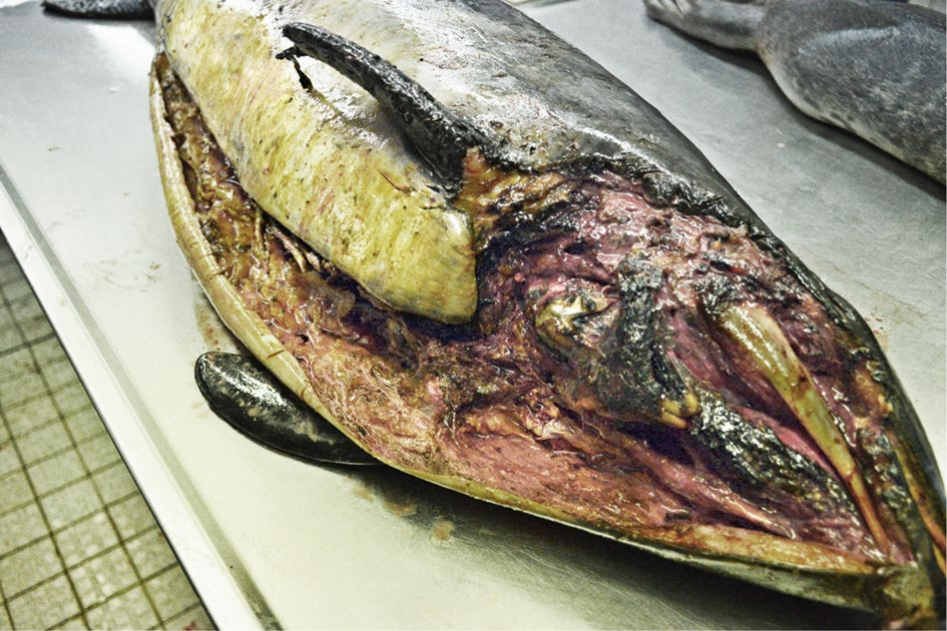
For grey seal predation untypical lesions in a porpoise carcass showing a potential knife induced lesion starting in the area of the head/throat and showing a smooth wound margin. In addition, signs of scavenging are present in the head area with considerable amounts of tissue removed and further manipulated blubber tissue in the area of the linear lesion.

When verifying the accuracy of the suggested decision tree by comparing the expert judgment with the results given by the tree, of the 250 assessed porpoises results from the tree matched the expert opinion in 96% of cases. As seen in seals, cases with a differentiating result often showed complex wound patterns; as for example, potential knife cuts in combination with clear signs of scavenging (e.g. Fig. 5).

When comparing the solely tree-based decisions to the expert opinion, only 50% of the differing decisions were more precautious (indicating that grey seal predation is less likely) than the expert judgement. This contrasts with the results shown for seals, where the tree-based decision was in most differing cases more precautious^14^.

### Anecdotal data

In addition to the cases for which a carcass was retrieved, we filed four cases of anecdotal reports of suspected grey seal predation of harbour porpoises recorded by professionals or semi-professionals in the field, but with no carcass retrieved for necropsy (Fig. 2).

## Discussion

When assessing the ecological status and the development of populations, one important factor to consider is the mortality rate and its underlying causes^15^. If the status of a population is deemed unsustainable due to high mortality rates, this information can then be used to develop and implement specific management measures, for example addressing the major causes of unnatural mortality^16^. With regard to the potential ecological relevance of the phenomenon of grey seal predation, it is therefore also important to have reliable estimates of harbour porpoise mortalities resulting from grey seal predation as one natural cause. To allow for a standardised assessment of lesions found in suspected grey seal predation cases, this study aims to summarise the knowledge that has been gathered to date.

The parameters described resemble the most commonly detected lesions in “definite”, “likely” and “fox” related cases of the 246 stranding records categorised as “suspicious” in terms of grey seal predation from the coasts of Schleswig–Holstein. With regard to grey seal predation, parameters 1–9 represent typical lesions, whereas the presence of parameters 10 and 11 is consistent with an interaction with a red fox.

Similar to lesions detected in seals, lesions in porpoises most often resemble puncture lesions in the skin and blubber (parameter 1). Yet, visually most striking is the commonly detected large tissue defect with straight, cut-like wound margins with often flaps of skin and blubber remaining only partly attached to the body (parameters 2, 5, 7). Missing blubber (parameter 4) is also recorded, either as reduced blubber thickness on the flaps of skin or as fully removed parts of blubber and skin. As has been described for seals^14^, the lesion most often originates in the cervical area (parameter 3). A difference that has been detected between the lesions in seals and porpoises is the rate of clear parallel running bite and / or scratch marks in the skin of the animals. Whereas this is rarely detected in seals^14^, most porpoises show respective marks (parameter 6). One probable explanation for this dissimilarity could be the different physical and morphological properties of the two types of skin. Seal skin is very dense and elastic; tearing and rupturing the skin requires a considerable amount of force^17^. Porpoise skin, however, is rather susceptible to applied mechanic force and puncturing or tearing it requires comparably little force^18^. These different mechanical properties might also be the reason why rake marks are found in the blubber (parameter 9) more often in seals (91% of likely cases^14^) than in porpoises (62% of likely cases). For seals, in the majority of cases, little to no skin is missing (skin missing in 49% of likely cases^14^), whereas in porpoises, a considerable number of cases (81% of likely cases) have been found where skin is missing (parameter 7). Grey seals have been described to mainly target the energy rich blubber tissue of their prey^11,14^. For the elastic and robust seal skin, this is done by scraping off the blubber with the teeth. As porpoise skin is fragile, we suggest that the blubber, including the skin, is more often fully removed by the grey seal and swallowed whole. If true, this may also influence the net energetic gain, which is acquired by the predator. Scraping of blubber tissue from seal skin will likely yield less tissue and cost more energy than tearing off whole pieces of blubber (including skin). Thus, it may result in a lower energetic gain. However, it is still unclear if the process of catching a porpoise in comparison to younger seals might also cost a considerably larger amount of energy, negatively influencing the net gain.

Similar to what has been described for seals, the avulsion of one or both scapulae (parameter 8) can be found and is also likely the result of the force applied when detaching the epidermis and blubber from the body of the prey^14^.

For porpoises, all nine suggested parameters were found in the definite case of grey seal predation. Parameters 1–5 showed a very high (100%) and parameter 6 a high rate (95%) of occurrence in likely cases. Parameters 7–9 occurred less frequently but were still found in > 60% of all likely predation cases. These high rates of occurrence throughout all parameters suggest that wound patterns found in porpoises are less variable than the patterns found in seals^14^. Whether this difference is a result of the different mechanical skin properties or if other factors are responsible, is beyond the scope of this study.

While for seals a skeletal trauma is used as an indicator of grey seal predation, for respective harbour porpoise cases, this is hardly ever (19% of likely cases) documented. In contrast, for porpoises, a skeletal trauma (parameter 10) is quite frequently detected in cases related to scavenging by foxes (46% of fox cases) where for example extremities can be manipulated^19^. As has been reported in seals^14^, the most often detected parameter in fox related cases is the ragged wound margin (parameter 11, 94% of the cases). Therefore, this can be seen as a good indicator of an interaction with a fox in porpoises. This is also supported by a definite case of fox scavenging, which was confirmed using genetic methods^20^. It needs to be stated though, that scavenging by birds can result in similar looking lesions, increasing the chance of misinterpretation. Scavenging by birds usually also leaves an irregular wound margin with extensive tissue loss. If parallel running lesions are present, the origin of the lesion can additionally be assessed by measuring the distances in between adjacent lesions and comparing them to published values of grey seal, fox and cetacean inter-teeth distances ^e.g.^^1^. This is especially important when differentiating between for example rake marks by dolphins, which have been documented in porpoises^21,22^ and marks induced by grey seals. Here, it can be useful to assess the pattern of inter-teeth distances with those of dolphins expected to be consistent in length, whereas for grey seals, variable distances are expected as the result of the polydont dental morphology^23^. Despite a lack of available data, a differentiation between grey seal claw-induced marks and dolphin rake marks should be possible, as distances between claws of a subadult / adult grey seal male are expected to be considerably larger than for dolphin inter-teeth distances.

Single puncture lesions, in turn, are not considered as a very good indicator despite being present in the definite and all of the likely cases. Mainly due to the susceptibility of the porpoise skin, such lesions can have many different causes (e.g. feeding by birds).

Whether a loss of muscle tissue can be attributed to grey seal predation or is largely caused by scavengers like gulls as has been suggested for seals^14^, is still not entirely clear. In German as well as bordering waters, no clear pattern prevails. Carcasses with mainly intact as well as fully removed muscle tissue have been documented ^c.f.^^13^. However, the reports by Stringell et al.^4^ suggest that not only the blubber tissue is targeted, but that there may also be some individual behavioural variation.

The findings and the resulting parameters described here are in line with wound patterns reported in earlier publications from other areas^1,6,7,13^. This shows that the documented wound patterns make a reliable set of parameters when assessing harbour porpoises carcasses potentially predated by a grey seal and should be used in future assessments.

As a complementary tool to the suggested parameters, corresponding to porpoises, we developed a decision tree with the aim of supporting a standardised and information-based decision-making process. Despite an accuracy in decision-making of 96% when using our data set, the example in Fig. 5 illustrates the limitations of such static tools when it comes to judging more complex cases. Furthermore, when comparing the suggestion given by the tree with the one made by the experts, in only 50% of unmatched cases, a rather precautionary judgement was made, bearing the risk of an overestimation of case numbers. Therefore, we recommend using the suggested tree only as an informational tool in supporting decision-making and final judgments should always be made by the responsible expert based on all available information.

In addition to cases for which the attack of a grey seal directly led to the death of the animal, interestingly, it seems not unusual that porpoises escape this predator. Several observations have been described in the literature^5,6,13,24^ and nine cases were documented in German waters (Figs. 1, 2). In order to be able to verify the origin of recorded teeth marks in porpoise skin, it is crucial to record marks in detail including their pattern, location and inter-teeth distances. Using the latter, for example, interactions with dolphins can potentially be excluded. Although there has been the odd case of a severely injured seal showing comparable lesions to what is associated with grey seal predation^14^, such high rates of escape cases as described for porpoises have not been reported.

Despite the co-occurrence of porpoises and grey seals in the Baltic, no case of grey seal predation on a porpoise can be confirmed by the presented results. It remains unclear whether grey seals in this area of the Baltic just don’t prey on porpoises or whether other factors like differences in behaviour (e.g. primary area of predation further offshore) are involved.

Some of the observed behaviour of grey seals when catching a porpoise can be directly linked to the detected lesions. For example, Stringell et al.^4^ as well as Bouveroux et al.^7^ described the grey seal acting as an ambush predator and attacking the porpoise from below using its jaws to catch and retain the prey. Lesions starting in the throat area (parameter 3) combined with parallel multifocal puncture lesions (parameter 6) resemble what would be expected as the result of such an attack.

Despite a lower rate of variability in detected wound patterns in porpoise carcasses, care should be applied when assessing lesions, as there is always the chance of other factors being involved. Therefore, if possible, a combination of data sources (necropsy results, genetic detection of predator DNA, indicators at the stranding site, eye witness reports, etc.) should be used in a systematic evaluation.

Future research should focus on continuing thorough investigations of stranded marine mammal carcasses in order to further update and refine the suggested set of parameters. Additionally, results of current as well as retrospective analysis of stranding data should be used to support an evaluation of the ecological relevance of this behaviour.

## Conclusion

As has been described for seals, during the pathological assessment of harbour porpoise carcasses, challenges can prevail when it comes to the differentiation of lesions induced by grey seals from other sources of trauma. Therefore, it is important to constantly refine sets of parameters deemed useful in this context as soon as new knowledge arises. For a thorough investigation, it is crucial to examine any carcass in detail using standardised parameters as well as to consider any source of information like reports on indicators at the stranding site or eyewitness reports. To increase the rate of standardisation during the decision-making process, tools such as the presented decision tree can be of help. In addition to the pathological assessment, the use of supporting methods such as the molecular verification of specific predator DNA is also highly encouraged^3,8,20^. The development of other complementary methods such as the histopathological assessment of the wound margin as an objective parameter would further increase the reliability of the results.

Future research should also focus on elucidating the—to date predominantly unknown—behavioural mechanisms behind this phenomenon, using observational and bio-logging techniques.

Despite the many questions that remain unanswered, it needs to be emphasised that up until today a solid knowledge base has been built by research around the North Sea and beyond. This was achieved in a joint effort to document and publish new findings with regard to the phenomenon of grey seal predation on marine mammals aiming at reliable future assessments of its ecological relevance.

## Methods

Carcasses were collected through the stranding network of Schleswig–Holstein, Germany^25,26^ and a necropsy was performed as described in Siebert et al.^26^. In contrast to seals, in German waters no case of a harbour porpoise being preyed on by a grey seal has to date been fully documented, comparable to what has been described in seals by van Neer et al.^11^. In order to validate the documented wound patterns, one case of grey seal predation which was confirmed using genetic methods and was therefore labelled a “definite” case was used^8^. Necropsies included age estimation, determination of sex, recording of weight and length as well as assessing the health status and cause of death. As previously described for seals, the decomposition state of the animal was rated by assessing the remaining intact parts of the body^14^. In most cases, detailed information on the stranding site, condition of the carcass when found, as well as any other useful information was recorded by local volunteers.

Of the 4463 stranding records available, cases of 1183 harbour porpoises with any recorded external wounds were rated as suspicious and selected for further investigations. Results were taken from the necropsy and stranding database of the Institute for Terrestrial and Aquatic Wildlife Research (ITAW), University of Veterinary Medicine Hannover (Büsum, Germany) and assessed retrospectively.

Cases, which were considered too decomposed for a thorough evaluation or data deficient, were categorised as “unknown” and excluded from the study.

Since the recognition of the predation phenomenon and the start of a specific research project in 2015, a detailed protocol was developed for recording potential cases of grey seal predation as part of the necropsy (see Fig. S1 in the supplementary material for the updated version based on the results presented here).

Necropsy results as well as available pictures and any additional information were evaluated in order to judge and rate assessed cases using six previously defined categories (definite, likely, possible, escape, unlikely and fox), Categorisation was carried out by experienced scientists depending on the likelihood of grey seal predation as an origin of documented lesions (Table 1). The resulting frequency of lesions was then in turn used to develop parameters to be considered in future pathological assessments.

A decision tree was developed using the data on presence / absence of suggested parameters as well as a combination thereof in given cases combined with the categorisation of likelihood resulting from the expert judgment. Verification of the reliability of the tree was conducted by comparing categorisation carried out by the expert with the solely tree-based suggestion.

Besides any cases where the carcass was retrieved and was available for necropsy, anecdotal data consisting of well-documented observational reports from Helgoland, Germany mostly recorded by professionals and semi-professionals and for which no carcass was retrieved, were filed and summarised. These cases were labelled “observation only”.

### Ethics approval and consent to participate

Carcasses used in this study were forwarded to the Institute for Terrestrial and Aquatic Wildlife Research for necropsy as part of the national stranding network and monitoring scheme. As all research was conducted on deceased animals, no ethics approval was required.

## Supplementary information

Supplementary file1

## Data Availability

All data generated or analysed during this study are included in this published article [and its supplementary information files].
