# Supplementary material for: Assessing harbour porpoise carcasses potentially subjected to grey seal predation
Source: Sci Rep. 2020 Oct 1;10:16345. doi: 10.1038/s41598-020-73258-y (PMC7530704; doi:10.1038/s41598-020-73258-y)
Supplement: Supplementary file 1 — Supplementary file1 [file 41598_2020_73258_MOESM1_ESM.pdf]

Assessing harbour porpoise carcasses potentially subjected to grey seal predation

Authors:

Abbo van Neer, Stephanie Gross, Tina Kesselring, Miguel L. Grilo, Eva Ludes-Wehrmeister, Giulia Roncon, Ursula Siebert

Supplementary Information

Table S1: Table showing 11 parameters, which have been added to the catalogue and are now routinely used for rating the likelihood of grey seal predation as origin of a trauma in porpoises.

**1**	**Puncture lesions:**<br>Often repetitive puncture lesions are present in the skin or blubber tissue.	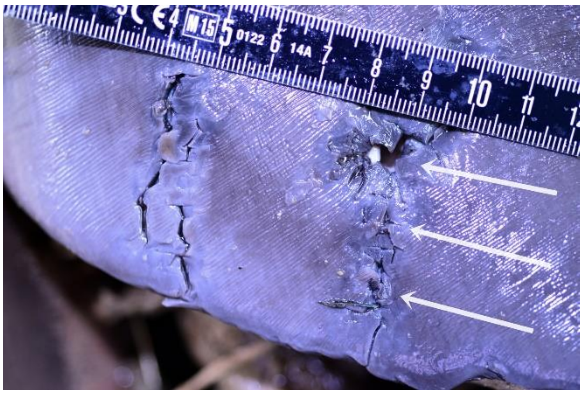
**2**	**Smooth wound margin:**<br>Due to the tearing of the skin, a smooth, cut like wound margin is present throughout large parts of the lesion.	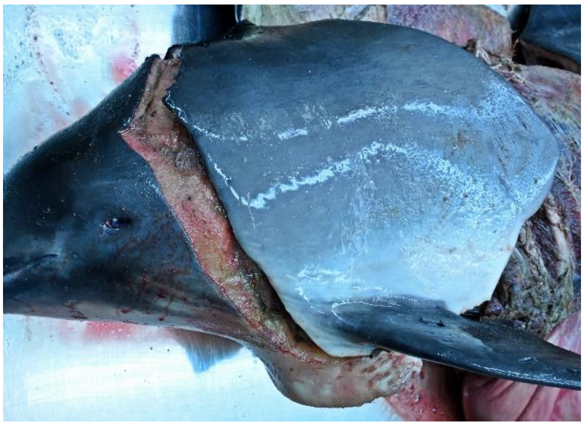

**3**	**Start of lesion in throat / head area:** The origin of the lesion lays on the ventral side of the neck or around the lower jaw / throat area.	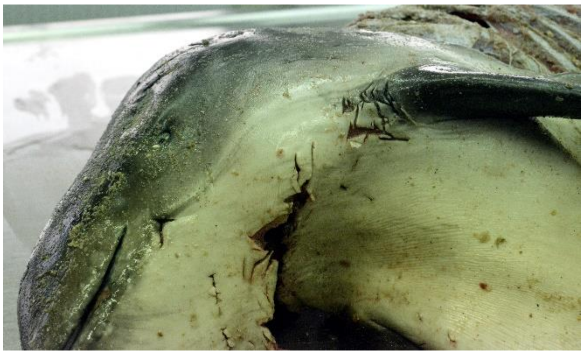
**4**	**Missing of blubber tissue:** Parts of the blubber tissue show signs of manipulation. Blubber depth along the fringes of the skin flaps is often in parts reduced and less than towards the middle areas.	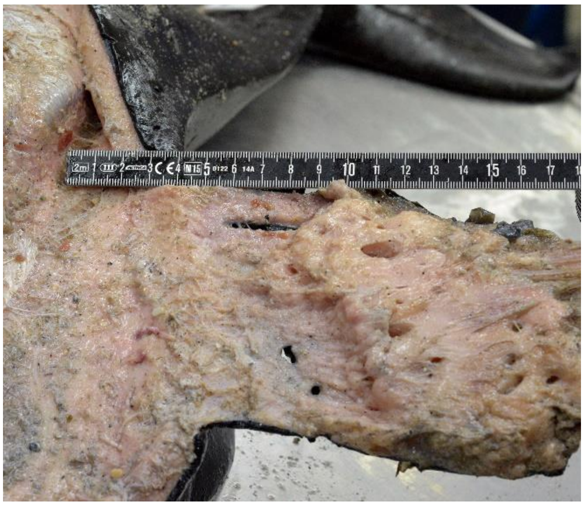
**5**	**Undermining / detachment of blubber:** Considerable parts of the blubber are detached from the underlying muscular tissue in large parts of the body area. Only in the areas around the caudal end of the body, the fluke, as well as the rostral part of the head, the skin and underlying tissue is often still attached.	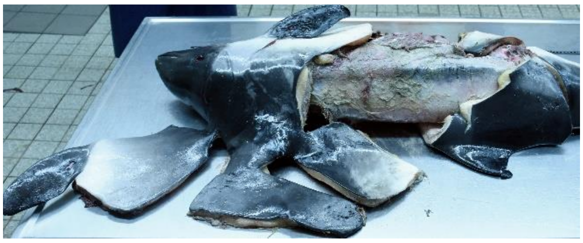

**6**	**Parallel bite / scratch marks:** Parallel running scratches resembling claw scratches or repetitive, parallel puncture lesions resembling bite marks are commonly observed. Bite marks in the area of the tail stock are usually found to be present bilaterally.	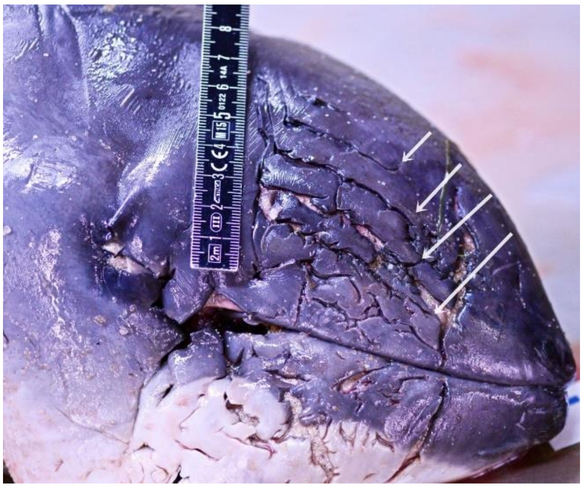
**7**	**Areas of skin are missing:** Larger areas of skin including the underlying blubber tissue can be missing.	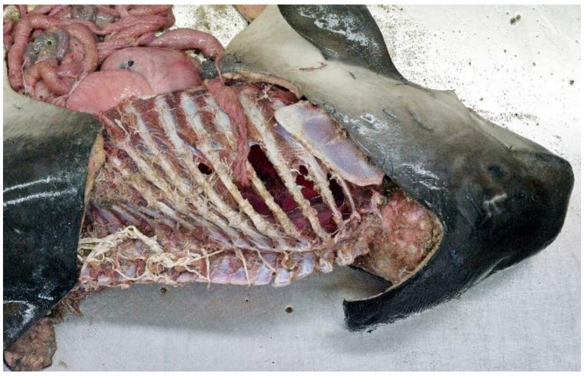
**8**	**Avulsion of one or both scapulae:** An avulsion of one or both scapulae due to the detachment of the skin and blubber (including the pectoral fins) can be present.	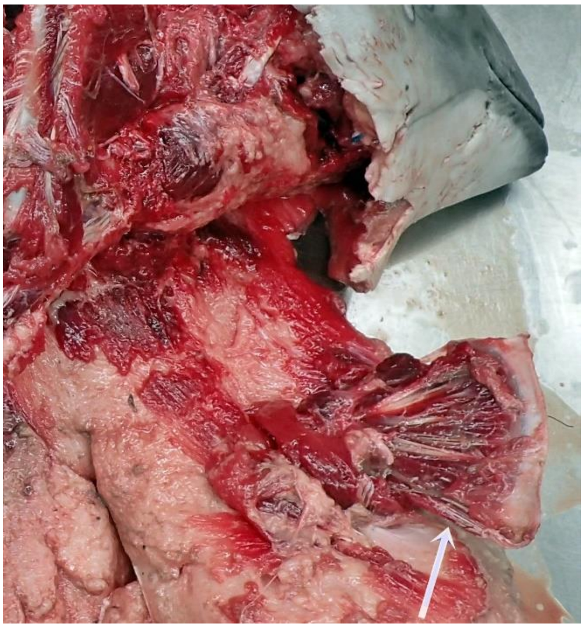

**9**	**Rake marks in blubber:**<br>Rake marks potentially as the result of the incisions by the teeth and / or claws are present in parts of the blubber tissue.	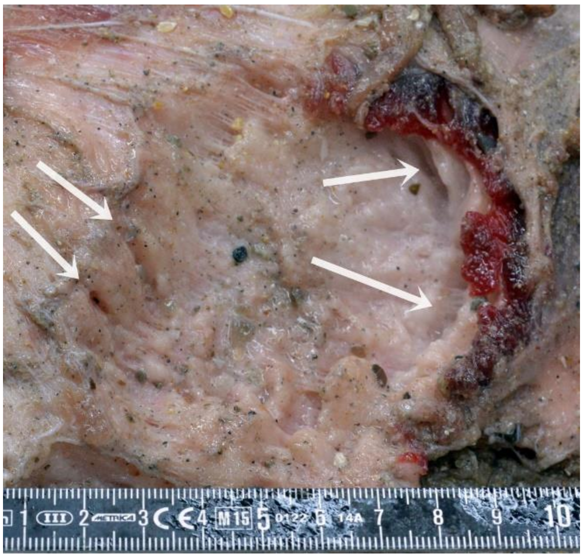
**10**	**Skeletal trauma:**<br>Fractures of bones with different severity can be present. Fractures / removal of parts of or whole extremities have been observed commonly in fox related cases. Also puncture-like fractures in the lower mandible and / or scapula have been observed commonly.	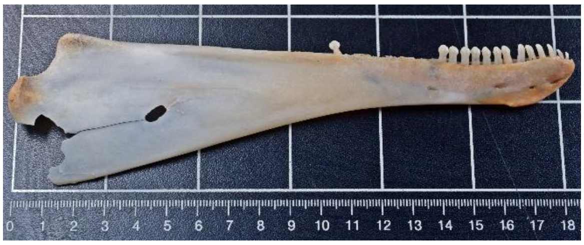
**11**	**Ragged wound margin (fully or in parts):**<br>Considerable parts of the wound margin have a ragged, uneven and not cut-like structure. This is a strong indication for an interaction with a red fox or other scavengers.	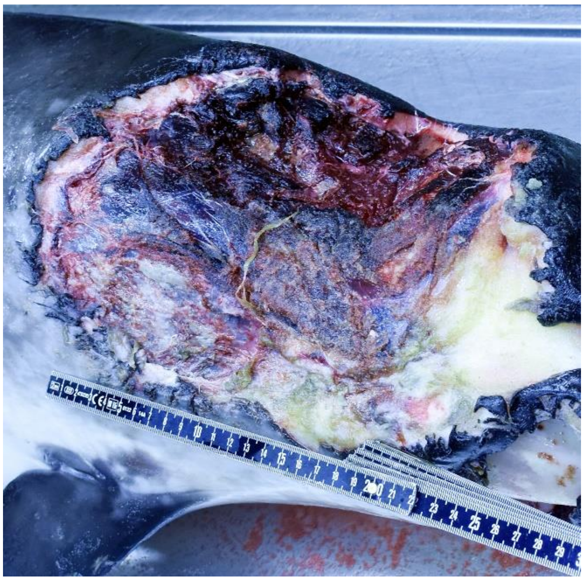

Figure S1: Newly developed protocol to be used for the documentation of suspected grey seal predation cases in its updated version based on the results presented here.

CETACEA-PROTOCOL Team:…………………………………………….

Prep-No................................ **Species**....................................... total length.................. cm

Date of retrieval........................... Prep-date............................ weight.......................... kg

Place of stranding.. Nr........................ sex m ◯ f ◯

Retrieved by...................................... Condition during delivery ☐ fresh ☐ frozen

Transponder / Tag number... **estimated age** ◯ juvenile ◯ subadult ◯ adult

Decomposition status ☐ (1-5)

Stranding Report

Pictures from stranding site: **O** yes **O** no

Animal tracks on or near site of stranding?

O terrestrial predator/scavenger **O** seal **O** none **O** no information

Prep.-No:_______________________

Documentation of lesions and sampling sites:

ventral

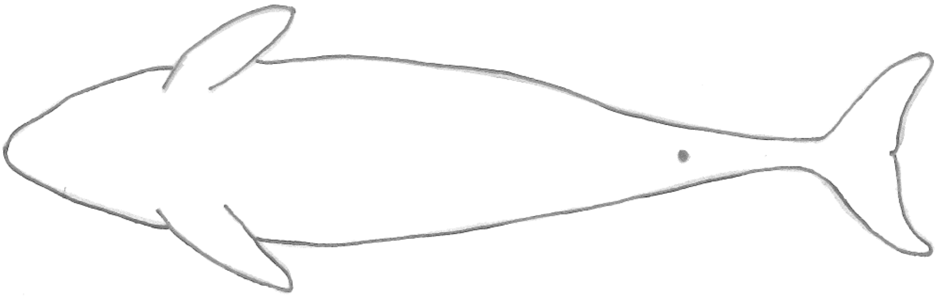

dorsal

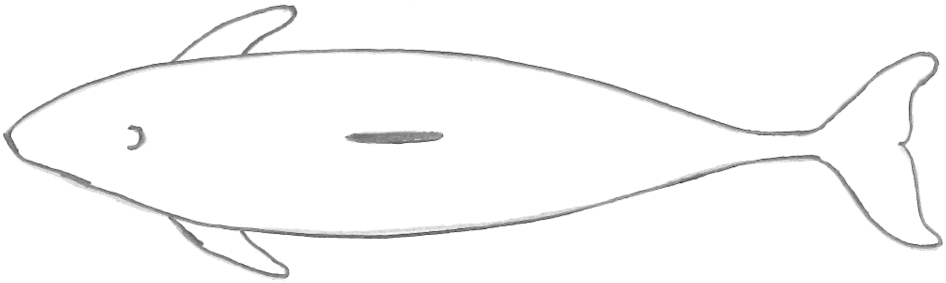

Right side of body:

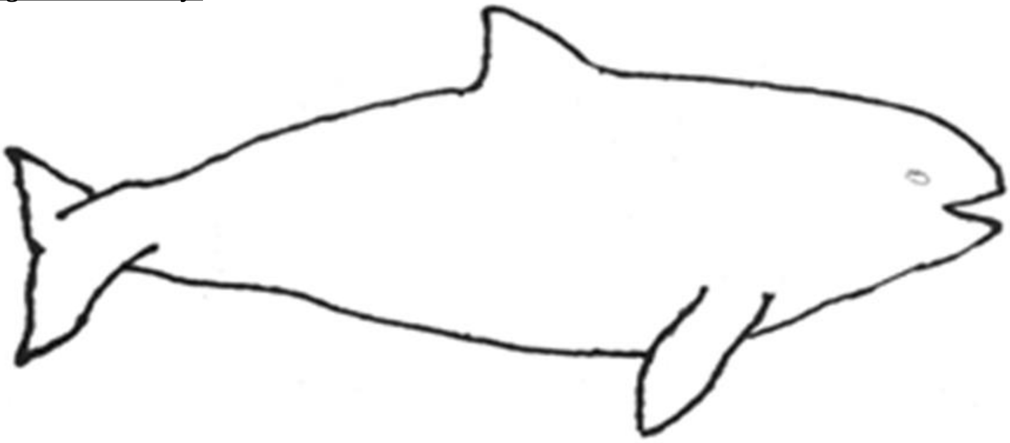

Left side of body:

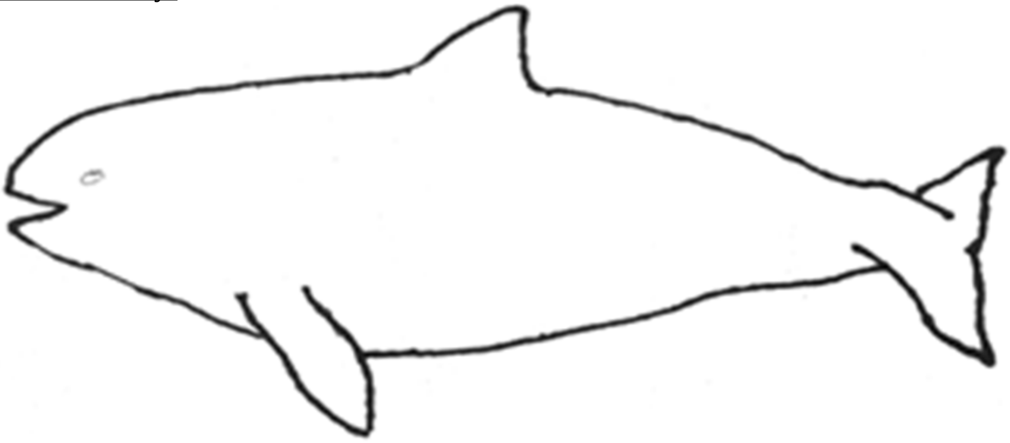

Prep.-No:______________________

Occurrence of Parameters:

No	Parameter	Absent	Present
1	Puncture lesions:<br>Puncture lesions are present in the skin or blubber tissue.	☐	☐
2	Smooth wound margin:<br>Due to the tearing of the skin, a smooth, cut like wound margin is present throughout large parts of the lesion.	☐	☐
3	Start of lesion in throat / head area:<br>The origin of the lesion lays on the ventral side of the neck or around the lower jaw / throat area.	☐	☐
4	Missing of blubber tissue:<br>Parts of the blubber tissue show signs of manipulation. Blubber depth along the fringes of the skin flaps is often in parts reduced and less than towards the middle areas.	☐	☐
5	Undermining / detachment of blubber:<br>Considerable parts of the blubber are detached from the underlying muscular tissue in large parts of the body area. Only in the areas around the caudal end of the body, the fluke, as well as the rostral part of the head, the skin and underlying tissue is often still attached.	☐	☐
6	Parallel bite and / or scratch induced lesions:<br>Parallel running scratches resembling claw scratches or repetitive, parallel puncture lesions resembling bite marks are commonly observed. Bite marks in the area of the tail stock are usually found to be present bilaterally.<br>➔ Measure distance between lesions!	☐	☐
7	Areas of skin missing:<br>Larger areas of skin including the underlying blubber tissue can be missing.	☐	☐
8	Avulsion of one or both scapulae:<br>An avulsion of one or both scapulae due to the detachment of the skin and blubber (including the pectoral fins) can be present.	☐	☐
9	Rake marks in blubber:<br>Rake marks potentially as the result of the incisions by the teeth and / or claws are present in parts of the blubber tissue.	☐	☐
10	Skeletal trauma:<br>Fractures of bones with different severity can be present. Fracture / removal of parts of or whole extremities have been observed commonly in fox related cases. Also puncture like fractures in the lower mandible and / or scapula have been observed commonly.	☐	☐
11	Ragged wound margin (fully or in parts):<br>Considerable parts of the wound margin have a ragged, uneven and not cut-like structure. This is a strong indication for an interaction with a red fox.	☐	☐
12	Scar tissue:<br>Scarred lesions are present resembling teeth or claw marks, e.g. parallel running healed rake marks. Marks on the tail stock are often bilaterally. This is an indicator for an earlier interaction with a grey seal, a respective escape of the individual and a subsequent (mostly) unrelated cause of death.<br>➔ Measure distance between scars!	☐	☐

Prep.-No:______________________

Photos yes ☐ no ☐

Detailed morphometry:

Measurements: (cm)
(1) .... Tip of snout - notch of fluke (= Total length)
(2) .... Tip of snout - rear rim of fin
(3) .... Tip of snout - front tip of blowhole
(4) .... Tip of snout - front tip of flipper
(5) .... Width of fluke
(6) .... Starting point of flippper – tip of flipper

Weight __________________________ kg

Girths (mm)
U1 ………. U4 ……….
U2 ………. U5 ……….
U3 ………. U6 ……….

Blubber thickness (mm) (with skin)
D2 ………. D3 ………. D4 ……….
L2 ………. L3 ………. L4 ……….
V2 ………. V3 ………. V4 ……….

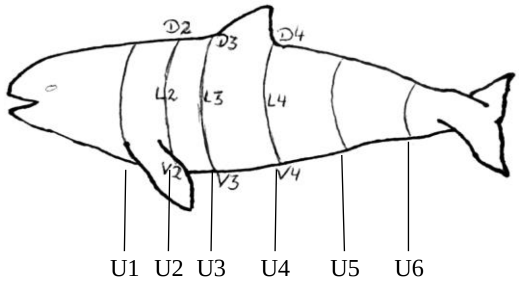

Nutritional Status ○ good ○ moderate ○ poor

Histological samples ☐

____

____

____

Bone samples ☐

Skeleton ○
Skull ○
Other ○ …………………………

Notes
